# Multi-level index construction method based on master–slave blockchains

**DOI:** 10.1038/s41598-024-54240-4

**Published:** 2024-02-19

**Authors:** Haolin Zhang, Su Li, Chen Liu, Guiyue Zhang, Baoyan Song, Junlu Wang

**Affiliations:** School of Information, Liaoning University, Shenyang, 110036 China

**Keywords:** Blockchain, Indexing, Sharding, Jump consistency hashing, Improved bloom filter, Electrical and electronic engineering, Mechanical engineering, Theory and computation

## Abstract

Master–slave blockchain is a novel information processing technology that is domain-oriented and uses efficient cryptography principles for trustworthy communication and storage of big data. Existing indexing methods primarily target the creation of a single-structured blockchain, resulting in extensive time and memory requirements. As the scale of domain data continues to grow exponentially, master–slave blockchain systems face increasingly severe challenges with regards to low query efficiency and extended traceback times. To address these issues, this paper propose a multi-level index construction method for the master–slave blockchain (MLI). Firstly, MLI introduces a weight matrix and partitions the entire master–slave blockchain based on the master chain structure, the weight of each partition is assigned. Secondly, for the master blockchain in each partition, a master chain index construction method based on jump consistent hash (JHMI) is proposed, which takes the key value of the nodes and the number of index slots as input and outputs the master chain index. Finally, a bloom filter is introduced to improve the column-based selection function and build a secondary composite index on the subordinate blockchain corresponding to each master block. Experimental results on three constraint conditions and two types of datasets demonstrate that the proposed method reduce the index construction time by an average of 9.28%, improve the query efficiency by 12.07%, and reduce the memory overhead by 24.4%.

## Introduction

The blockchain technology utilizes a blockchain data structure to store and verify data, ensuring secure transmission and access through cryptography^1^. It possesses the characteristics of high credibility^2^, traceability, and decentralization^3^, effectively addressing the issue of trust in third-party data storage^4^. However, as blockchain technology advances and various industries accumulate vast amounts of data, the traditional single chain blockchain system proves inadequate for increasingly complex application scenarios. Consequently, master–slave blockchains (MSBC) structures such as Spark Chain have garnered attention from experts and scholars alike, finding extensive applications in security^5,6^, fog computing^7^, industrial internet of things^8^. Master–slave blockchains typically consist of a master chain comprising master blocks connected to slave chains composed of slave blocks. The connection between each master block and slave block is established using their respective hash values from previous blocks. Furthermore, unique hash values are employed to map the master chain with its corresponding slave chain.

This MSBC structure enables effective handling of intricate classification scenarios. For instance, within the financial sector, MSBCs facilitate the construction of company-specific blockchain systems that record financial activities. The master chain stores information pertaining to financial institutions while their transaction events, financial activities, and other relevant data are stored in corresponding slave chains. Through consensus mechanisms like HotStuff^9^and Algorand^10^, tampering with this data becomes impossible.

However, with the advancement of blockchain technology and the continuous accumulation and expansion of data in various industries, there is a constant need to update information on the chain. This results in disorderly storage of updated information at the same location, leading to low query efficiency^11^ and long traceability time^12^ in master–slave blockchain systems. Therefore, establishing an efficient and dynamically maintainable index structure for master–slave blockchains still poses challenges.

Firstly, existing blockchain index structures are primarily optimized for single-chain structures, which enhances query efficiency in such systems. Consequently, single-chain structured blockchains with optimized indexes exhibit better traceability^13^. Although applying these optimized index structures significantly improves query efficiency compared to original blockchain systems, their application to master–slave blockchains reduces the speed of indexing as demonstrated in subsequent experimental sections of this paper.

Secondly, constructing indexes consumes considerable time that directly impacts the query efficiency of master–slave blockchain systems^14^. For instance, if unoptimized, a master–slave blockchain with N additional child chains compared to a single-chain structured blockchain will require at least (N + 1) times more time for index construction. Furthermore, as the structure of a master–slave blockchain changes over time, its memory overhead for building traditional indexes increases when compared to single-chain structures.

To solve the above issues, this paper proposes a multi-level indexing method based on master–slave blockchains (MLI). The main contributions of this paper include the following:

1. This paper proposes a multilevel index structure, for the master–slave blockchain architecture to address the issues of significant time overhead and high memory consumption associated with traditional indexing research. It aims to enhance the query efficiency of the master–slave blockchain system.
2. To achieve index structure, this paper introduces a slicing algorithm that efficiently constructs the multilevel index for the master–slave blockchain structure and accelerates query processing speed. Prior to constructing the multilevel index, preprocessing is performed on the entire master–slave blockchain structure to reduce time overhead.
3. In response to low query efficiency and lengthy traceability time in existing blockchain indexes, this paper leverages both the structure of a master–slave blockchain and designs a jump consistency hash algorithm on the master chain. Additionally, an improved Bloom filter-based index construction method is proposed for optimizing column-based selection function on slave chains while providing an index query approach.
4. Compared with the existing methods on different constraints and data sets, the effectiveness of the proposed method is verified. After verification, it can be known that the MLI method proposed in this paper has the index construction time optimized by about 10.71% compared with the existing methods.

## Related work

At present, many scholars have conducted in-depth research on the index construction problem of blockchain and achieved certain research results.

Vikram Nathan et al.^15^ introduces Flood, a multi-dimensional memory read-optimized indexing method that dynamically adjusts to specific datasets and workloads by optimizing both the index structure and data storage layout. However, it lacks the capability to detect significant changes in query distribution, necessitating periodic evaluation of query costs for layout adjustments.

Huang et al.^16^ presents EBTree, a Level B Tree-based indexing method designed for real-time top-k, range, and equivalence searches on Ethernet blockchain data. However, its drawback lies in storing index nodes separately in Level DB, adversely affecting query efficiency due to node size considerations.

Andreas Kipf et al.^17^ proposes RS, a single-pass learning index method that efficiently constructs indexes in a single pass of sorted data using only two datasets. While friendly to most datasets, RS experiences performance degradation as the dataset size increases.

Xing et al.^18^ introduces SCATC, a subchain account transaction chain-based indexing method that divides the transaction chain into subchains with hash pointers connecting each subchain to the account branch node in the last block. It optimizes query efficiency for long account transaction chains but falls short in ensuring data privacy as it only addresses plaintext queries.

Noualhamdi et al.^19^ presents ChainLink, a scalable distributed index method for large time series data featuring a 2-tier distributed index structure and single-channel signature hashing. However, its limitation lies in the need for local data reorganization, compromising data security.

Gao Yuanning et al.^20^ proposes Dabble, a scalable learning index model based on the middle layer that utilizes the K-means clustering algorithm to divide dataset regions. It employs neural network learning for predicting data locations. However, Dabble exhibits poor timeliness in dataset updates, and the choice of K significantly impacts model accuracy.

In conclusion, this paper conducts a comprehensive investigation into the index construction of master–slave blockchains. Addressing the limitations of current indexing methods and taking into account the space–time complexity and data security associated with index construction, a multi-level index construction method for master–slave blockchains is introduced.

## Blockchain slice method based on master–slave structure

To achieve efficient index construction and query processing of the structure of the master–slave blockchains, firstly, the whole master–slave blockchains are sliced based on the characteristics of the master chain, and each segment is given a weight. Based on this, the segmentation weight matrix of the whole master–slave blockchains structure is constructed, and the number of nodes in the slice is determined based on the weight matrix, which provides support for the indexing of the master chain and slave blockchain.

### Construction of weight matrix

Let $x$ represent the number of nodes in the master–slave blockchains. The master–slave blockchains are segmented into $y \, (y \ll x)$ slices, where the *i*-*th* slice is denoted as $f_{i} (i = 0,1,2, \cdots ,y - 1)$, and each slice is assigned a weight $\omega_{i}$. The determination of slice weight is contingent upon three dimensions: node load, node credit, and network quality. Specifically, node credit and network quality exhibit a positive correlation with slice weight, while node load demonstrates a negative correlation. The weights associated with each dimension are established based on the optimal experimental results ratio.

#### Definition 3.1

(*Node Load*): Node load is the amount of work $L = \left\{ {l_{1} ,l_{2} , \ldots ,l_{i} , \ldots ,l_{n} } \right\},l_{i} \in R$ that the blockchain system assigns to any node. Where $l_{i}$ is the number of tasks assigned to the node and $n$ is the number of nodes.

#### Definition 3.2

(*Node Credit*): Node credit is the accumulation of contribution value $C_{i}$ and damage value $H_{i}$ of nodes in the blockchain system when nodes participate in block generation, verification and synchronization activities in the system. Here, $H_{i}$ is a negative value and $i$ denotes the number of nodes.

#### Definition 3.3

(*Network Quality*): Network quality is the evaluation metric of the network layer when nodes in the blockchain system participate in block generation, verification, and synchronization activities. Among them, node credit and network quality are positively correlated with fragment weight, node load is negatively correlated with fragment weight, and the weight of each dimension is determined by the proportion with the best experimental effect.

Before calculating the slice weight, the units of the above three dimensions are not uniform, so normalization is needed. Among them, the normalization formula of node load is:

1 $$d_{ij} = \left\lfloor {\frac{1}{{\log (d_{ij}^{\prime } + 1) + 1}} \times \frac{x}{y}} \right\rfloor$$

The normalization formula of node credit and network quality is:

2 $$d_{ij} = \left\lfloor {\frac{{d_{ij}^{\prime } + 1 - min}}{max + 1 - min} \times \frac{x}{y}} \right\rfloor$$

The weight of the *i*-*th* slice is $\omega_{i}$, and the calculation formula is shown in formula (3).

3 $$\omega_{i} = \sum\limits_{j - 1}^{3} {\omega_{ij} d_{ij} }$$

$d^{\prime}_{ij}$ in formulas (1) and (2) represents the original values of node credit and network quality, $d_{ij}$ is the normalized value, *max* and *min* are the maximum and minimum values of this component, respectively. The $\omega_{ij}$ in formula (3) represents the weight of each component.

Let each element of the two-dimensional weight matrix ***M*** be composed of slice weights. After the weight of each slice is obtained, a two-dimensional weight matrix ${\mathbf{M}}_{p \times q}$ is constructed by using each slice’s weight $\omega i$ (where $p \le \sqrt y$, *p* and *q* are integers). For any slice *f*, there is $M[{f \mathord{\left/ {\vphantom {f p}} \right. \kern-0pt} p}][f\% q] = \omega_{f}$, and the empty position in the matrix is set to 0.

### Determining the number of nodes in a slice based on weight matrix

Determine the number of nodes in each slice based on the weight matrix in Sect. 3.1. Firstly, the slice weights in the matrix are linearly normalized. Secondly, all normalized slice weights are dispersed in proportion, and the interval of discrete proportional weights is set as [1, *Q*]. The final slice proportional weight *Q*-1 is obtained, that is, the slice will correspond to *Q*-1 nodes.

Number the nodes in the order of {0, 1, 2, …, $\sum\limits_{i}^{x - 1} {\omega_{i} - 1}$}, and the node number corresponding to the *kth* slice is [$\sum\limits_{i = 0}^{k} {\omega_{i} }$, $\sum\limits_{i = 0}^{k + 1} {\omega_{i} } - 1$]. After obtaining the node number, the slice number can be obtained by looking up the table. Assuming that the proportional weight of the slice obtained after normalization and discretization is 7, the slice will correspond to 7 nodes, and the nodes will be numbered in the order of {0, 1, 2, …, 6}.

Among them, the linear normalization formula is:

4 $$\omega ^{\prime}_{i} = \frac{{\omega_{i} - \omega_{min} }}{{\omega_{max} - \omega_{min} }}$$

$\omega_{min}$ in formula (4) is the minimum value of all slice weights, $\omega_{max}$ is the maximum value, and $\omega ^{\prime}_{i}$ is the normalized slice weight.

Based on the weight matrix, a weighting matrix-based slice algorithm (WMBS) is proposed, which can quickly map the blockchain slice to nodes through the Jump Search algorithm and realize the slice of the structure of the master–slave blockchain. Enter the key value of the node key, random number *r*, and weight matrix, and call the jump search algorithm to map the nodes and slices one by one. The *key* is the key value of a 32-bit node, which is the unique identifier of the node. It is created when the node joins the blockchain and consists of an 8-bit fragment address code and a 24-bit node random code. *r* is a random number evenly distributed in the interval of [0, 1], which is generated by calling a linear congruence random number generator. WMBS algorithm is shown in algorithm 1.

**Figure:**
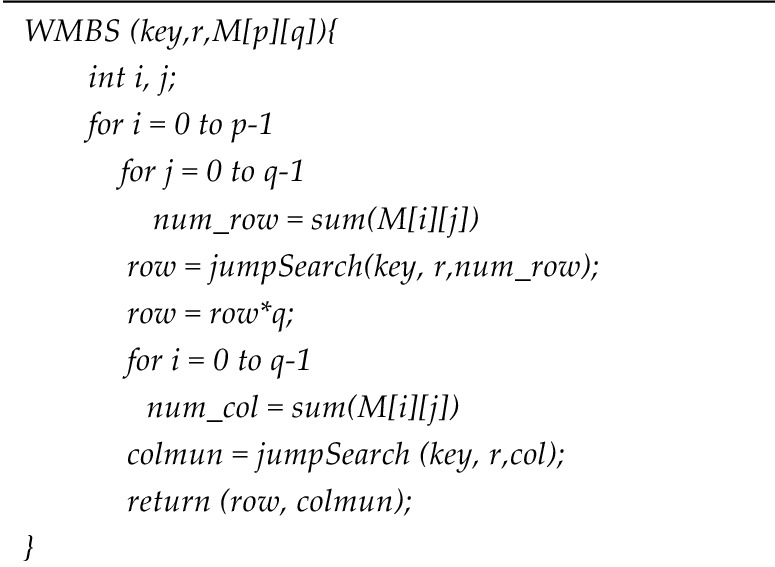
**Algorithm 1**. WMBS Algorithm

To optimize the path of searching nodes in a slice, which is lower than the time complexity of a linear search algorithm, this paper proposes a Jump Search algorithm, as shown in algorithm 2.

**Figure:**
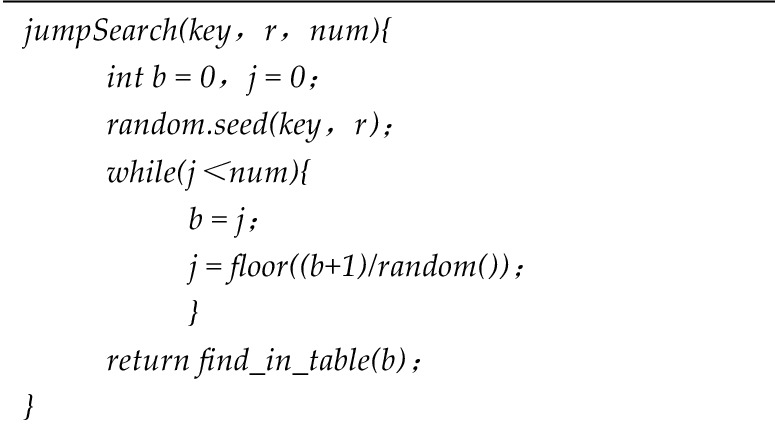
**Algorithm 2**. JumpSearch Algorithm

Where num is the number of rows or columns in the two-dimensional weight matrix. WMBS algorithm counts the number of columns in the weight matrix. First, it searches the key values of nodes in each row with the Jump Search algorithm to obtain the row number, then counts the number of columns in this row, and calls the Jump Search algorithm in each column to determine the column where the node is located, and finally returns the row and column number of the node.

## Methods for constructing multi-level index

**Figure 1:**
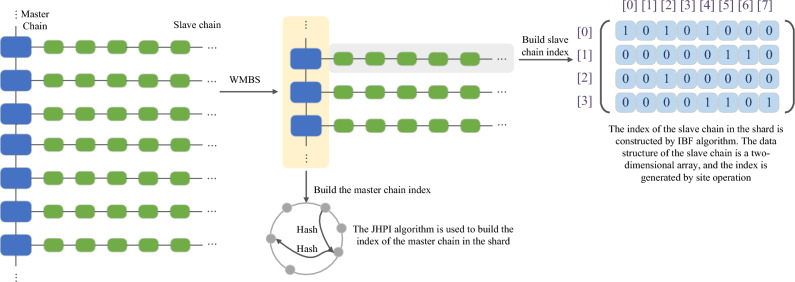
Multi-level index construction schematic.

Due to the variance in data scale and information types stored within the master chain and slave chain of master–slave blockchains, the application of the Weight Matrix-Based Slicing (WMBS) algorithm results in the need for a multi-level index construction method. This proposed method is based on the combination of the Jump Consistency Hash algorithm and an improved Bloom filter, tailored to meet the distinct query requirements of both the master chain and slave chain. The schematic representation of the multi-level index construction process is depicted in Fig. 1.

### Construction of master chain index based on jump consistency hash

Based on the characteristics of data stored in the master chain, this paper introduces the jump consistent hash algorithm and proposes a Jump Consistent Hashing-based Master Chain Index Construction method (JHPI) to expedite the construction of the master chain index. The process involves determining the number of index slots based on the nodes in each master chain slice. Subsequently, the key value of each node is established using the hash value of the master chain stored data. Finally, inputting the key value of each node along with the number of index slots yields the master chain index as output.

In the event of a change in the number of nodes within a slice, nodes undergo a jump in the index, resulting in the remapping of certain nodes. Let the hash mapping function responsible for this jump be denoted as *ch(key, num_buckets)*, where key represents the key value of nodes and num_buckets is the number of slots. The following mappings occur:

1. When *num_buckets* = *1*, indicating a single slot, all keys map to this slot, i.e., *ch(key, num_buckets)* = *0*, and all nodes are allocated to slot number 0.
2. When *num_buckets* = *2*, 1/2 nodes reside at *ch(key, num_buckets)* = *0*, while K/2 keys undergo remapping to *ch(key, num_buckets)* = *1*, resulting in a jump to slot 1.
3. When the number of slots changes from *n* to *n* + *1*, the slot for the node with *n/(n* + *1)* remains constant, denoted as *ch(key, num_buckets)* = *n-1*, and *1/(n* + *1)* keys necessitate remapping to *ch(key, num_buckets)* = *n*.

**Figure 2:**
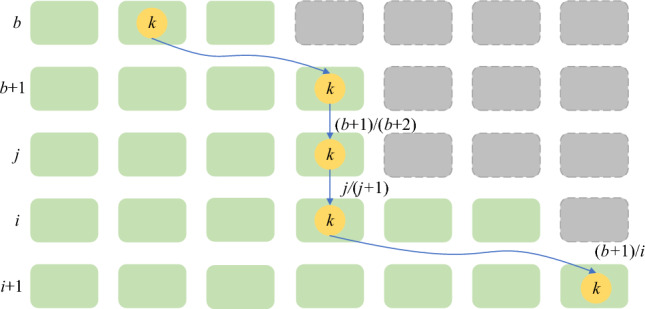
Diagram of node remapping.

The variable 'b' signifies the result of the last jump of the node, and 'j' represents the outcome of the subsequent jump. A schematic illustration of node remapping in response to changes in the number of nodes within a slice is presented in Fig. 2.

According to the analysis of Fig. 2, for any $i(i \in [b + 1,j - 1])$, the probability that the number of nodes does not jump is shown in formula (5).

5 $$P(i) = \frac{b + 1}{{b + 2}} \times \frac{b + 2}{{b + 3}} \times \cdots \times \frac{i - 1}{i} = \frac{b + 1}{i}$$

Take a uniformly distributed random number *r* in the interval of [0,1], and get it from formula (5). When $r < {{(b + 1)} \mathord{\left/ {\vphantom {{(b + 1)} r}} \right. \kern-0pt} r}$, the node will jump to *j* the upper bound of *i* is ${{(b + 1)} \mathord{\left/ {\vphantom {{(b + 1)} r}} \right. \kern-0pt} r}$. Since there is $j \ge i$ for any *i*, then $j = floor({{(b + 1)} \mathord{\left/ {\vphantom {{(b + 1)} r}} \right. \kern-0pt} r})$. The construction method of the master chain index based on jump consistent hash is shown in algorithm 3.

**Figure:**
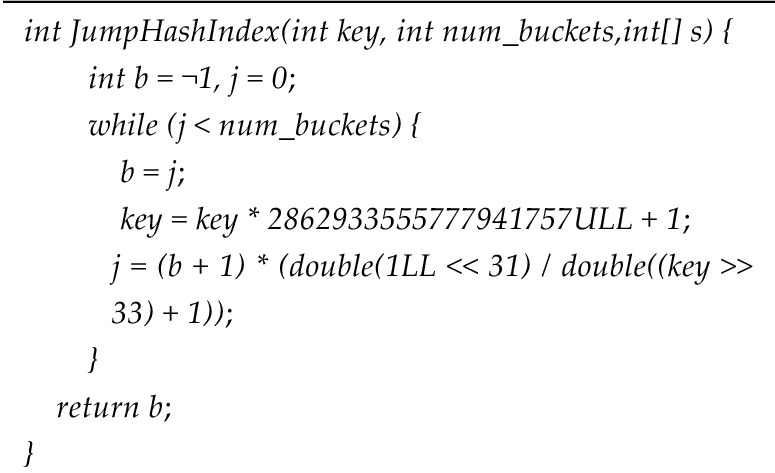
**Algorithm 3**. JHPI Algorithm

The JPHI algorithm takes the node *key*, the number of slots *num_buckets*, and the empty main chain index (s) as inputs. Initially, a *while* loop is executed, and as long as the number of slots in the last slot expansion before the jump is less than the total number of slots, the algorithm proceeds. During this process, the number of slots in the last slot expansion before the jump is assigned to variable *b*. Subsequently, the node key, combined with a pseudo-random number, generates the node key for the next operation. The combination of *b* and the node key undergoes an operation followed by rounding down to determine the node's position number, which is then input into the main chain index 's'. This process is repeated, generating a new node key value for subsequent operations. 'b' and the node key value undergo a similar operation to derive the node's position number, which is once again input into the main chain index *s*. This iterative process continues until the desired number of slots is achieved. Finally, the completed main chain index is output, concluding the index construction process.

### Construction of slave chain index based on improved bloom filter

In the process of constructing the slave chain index, the data stored in the dependent chain has the characteristics of large-scale and heterogeneous sources. Therefore, this paper reconstructs the data structure of the Bloom filter, proposes a column-based selection function, and realizes the slave chain index construction method (IBF) based on the improved Bloom filter.

Firstly, the data structure of the slave chain index based on the improved Bloom filter is constructed as a two-dimensional array *A*[*p*][*q*], where $p = 2^{n} ,\;n \in {\mathbb{N}}$, and the data length in* q* is $l_{q}$, assuming that $l_{q}$ = 32/64 bits. The value is determined by the cache line length of general registers in the CPU to reduce memory access and improve query performance. Let the* K* hash functions of the improved Bloom filter be Hash(*key*), where the *key* is the key value of the node, and let the length of the elements that can be stored in an improved Bloom filter be *len*, and the calculation result of *len* is shown in formula (6).

6 $$len = p \times q = 2^{n} \times l_{q}$$

After the data structure of the index is constructed, the index of the slave chain corresponding to each main block is constructed, and the specific steps are as follows:

- Step1: Use the function of selecting columns, first map the element to the corresponding column, and the element will be at the position of the corresponding column;
- Step2, obtaining the locus through *K* hash functions;
- Step3: Set the corresponding site to 1, and the rest sites to 0.

Within this process, the column selection function in Step 1 introduces additional computational overhead. To mitigate this overhead, optimization of the hash function is pursued, and the transaction hash value stored in the slave chain is derived using the SHA256 hash function. Consequently, the column selection function in Step 1 undergoes optimization based on the SHA256 hash function, and it can be obtained through a modular operation. The selection column function in Step1 can be expressed as:

7 $$qv = v\% 2^{n}$$

In Step2, the *K* hash functions are composed of *K* bitwise AND operations, which can be expressed as:

8 $$pv^{i} = v\& \sum\limits_{j}^{{i \times \frac{k^{\prime}}{k} - 1}} {2^{i - 1} } (0 < i \le k)$$

The *v* in formula (7) is the element in the Bloom filter and $qv$ is the column number obtained after column selection. After obtaining the column in which the element is located, it is determined that the element will be limited to the corresponding column during construction and query. The $pv^{i}$ in formula (8) is the row number obtained by *K* times of operation of formula (8) in the column after the column number is obtained, that is, the corresponding position, and $k^{\prime}$ is the array length in the general Bloom filter. The slave chain index construction algorithm based on the improved Bloom filter is shown in Algorithm 4.

**Figure:**
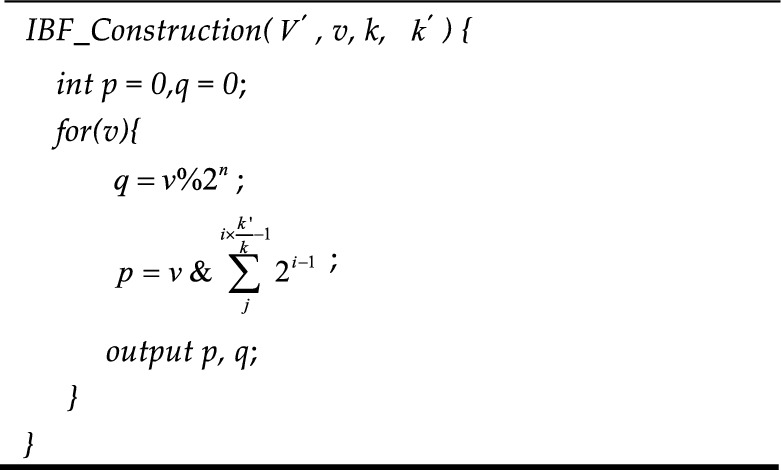
**Algorithm 4**. IBF_Construction

After constructing the index of the slave chain, an index query algorithm of the slave chain based on an improved Bloom filter is proposed according to the key values of nodes on the slave chain and the selected column function, as shown in algorithm 5.

**Figure:**
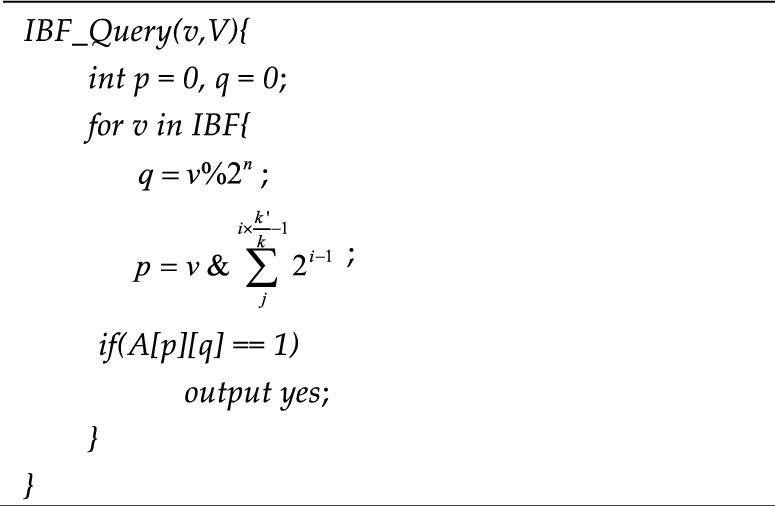
**Algorithm 5**. IBF_Query

### Example

**Figure 3:**
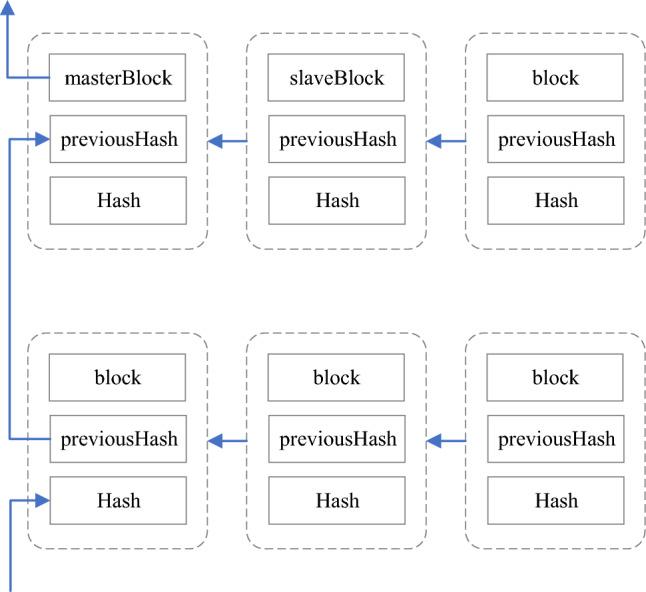
Financial master–slave blockchain architecture.

Consider the instantiation of master–slave blockchain indexing within the financial domain. In financial applications, a master–slave blockchain is employed to establish an enterprise blockchain system dedicated to financial activities. In this context, the master chain is responsible for storing attribute information pertaining to the financial enterprise, while the associated slave chain houses data related to transaction events, financial activities, and similar elements. The architectural representation of this blockchain structure is depicted in Fig. 3.

**Figure 4:**
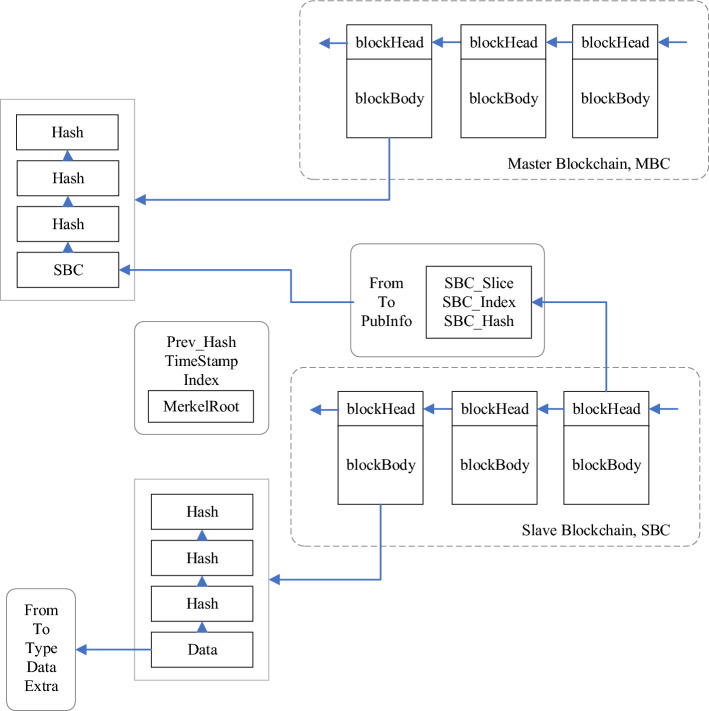
Linking relationship between master chain blocks and slave chain blocks.

Within the financial master–slave blockchain architecture, the linkage among blocks occurs through the hash value of the preceding block stored within the block body. This linkage is established for both the blocks within the master chain, between blocks within the slave chain, and between blocks of the master chain and their corresponding counterparts in the slave chain. Figure 4 illustrates the linking structure between the blocks of the master chain and their corresponding blocks in the slave chain.

In the initial phase, prior to constructing the multilevel index, preprocessing of the master–slave blockchain structure is imperative to enable the efficient establishment of the multilevel index. Leveraging the master chain, the entire master–slave blockchain structure undergoes slicing, and slice weights are assigned to formulate the slice weight matrix. This matrix, in turn, determines the node count within each slice. The computation of the number of nodes in each slice involves constructing a slice weight matrix based on the master chain and applying slice weights. Subsequently, a blockchain slicing algorithm, named Weight Matrix-Based Slicing (WMBS), is proposed.

Following this preprocessing, the modified master–slave blockchain structure is employed to construct multilevel indexes, adopting distinct approaches based on the unique characteristics of the data stored on the master and slave chains. On the master chain side, a Jump Consistency Hash algorithm is introduced, giving rise to a method termed Jump Consistency Hash-Based Master Chain Index Construction (JHPI), designed for rapid master chain index construction.

For the construction of the slave chain index, a reconstruction of the Bloom filter data structure is undertaken, coupled with the introduction of a column-based selection function. An innovative slave chain index construction method, denoted Improved Bloom Filter (IBF), is then proposed.

## Experiments

The experimental environment of this paper is 16 servers with 1 T storage space, 8G RAM, and a 4-core CPU. The servers communicate with each other through a high-speed network, and each server is equipped with an ubuntu 18.04 operating system. Two different data sets are used for experimental verification. The first data set is the first 3,000,000 blocks in the public Ethereum network, and there are 15,362,853 transactions in the data set. The second data set is the Lognormal artificial data set. Lognormal data set samples 5 million pieces of non-duplicate data according to lognormal distribution (mean value is 0, variance is 2). In this section, this paper will verify the high efficiency and low memory advantages of MLI from three aspects: index building time, query time, and memory consumption.

### Selection of slice weight proportion

**Figure 5:**
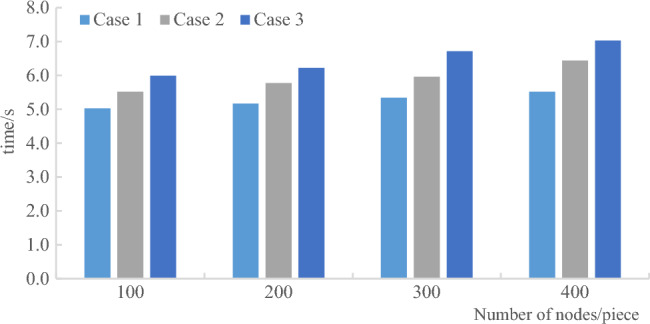
Slice weight ratio.

In the slice preparation phase, servers are tasked with constructing 10 slices, where each server creates a node and assigns it to the corresponding slice. The node capacity within each slice is fixed at 500 nodes. For varying node quantities (100, 200, 300, 400), the slice weight is determined by the ratios of node load, node credit, and network quality across three dimensions, specifically set at 3:3:4, 4:3:3, and 5:2:3. Figure 5 illustrates the experimental results.

From the experimental results in Fig. 5, when the number of nodes in the same, the experimental effect of case 1 is the best, and as the proportion of node load dimension increases, the slice time increases. With the increase of the number of nodes, the time of cases 2 and 3 increases, while the time of case 1 increases little, and the time stays at about 5 s. Therefore, when the blockchain is segmented according to the master blockchain, the slice weight of node load, node credit, and network quality is 3: 3: 4.

### Comparison of index construction time

To verify the efficiency of the MLI method proposed in this paper in terms of index construction time, the EBTree method with improved blockchain structure and Dabble model method with the neural network will be compared respectively. In the EBTree method, the capacity of internal nodes is set to 128, and the capacity of leaf nodes is set to 16. The value of k in Dabble model is 100, and the number of nodes in a slice of the MLI method is set to 100. In this section, the experiment will be divided into three specific situations as shown in Table 1.

**Table 1:** Index build time comparison.

Dataset one			Dataset two		
MLI	EBTree	Dabble	MLI	EBTree	Dabble
Case 1					
The slave block data is empty, and the number of slave blocks is 500, 1000, 1500, and 2000			The slave block data is empty, and the master block store 500, 1000, 1500, and 2000 pieces of data		
Case 2					
The master block data is empty, and the number of master blocks is 500 k, 1000 k, 1500 k, 2000 k, 2500 k, and 3000 k respectively			The master block data is empty, and the slave blocks stores 20 k, 40 k, 60 k, 80 k, and 100 k pieces of data		
Case 3					
The data of master and slave blocks are not empty, the number of master blocks is 500, 1000, 1500, 2000 respectively, and the number of slave blocks is 500 k			The data of master and slave blocks are not empty. The master block stores 500, 1000, 1500, and 2000 pieces of data, and the slave block stores 20 k pieces of data		

**Figure 6:**
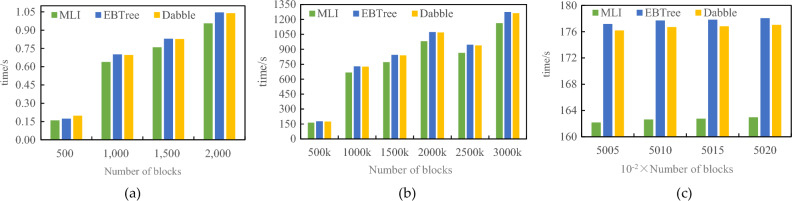
Index build time comparison 1.

**Figure 7:**
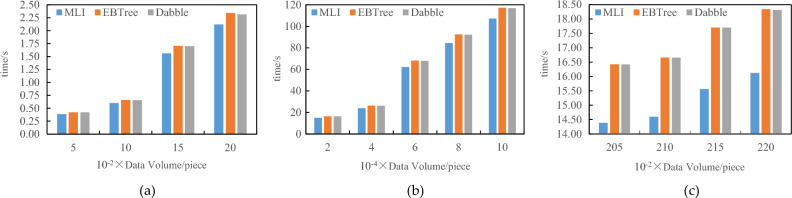
Index build time comparison 2.

As shown in Table 1, the experimental results of index construction time comparison are shown in Figs. 6 and 7.

As can be seen from Figs. 6 and 7, with the increase in data volume, compared with the existing methods, the index construction time of MLI is optimized by average of 9.28%.

### Comparison of query time

In this experimental section, both the indexes and data are initially loaded into memory. To assess the query performance of the Multilevel Index (MLI) method, this paper conduct a comparative analysis of query response times using datasets of varying sizes and under different query conditions.

#### Query response time comparison for large-scale datasets

**Figure 8:**
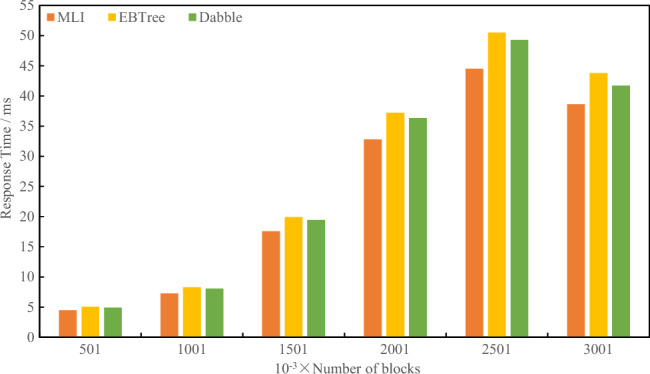
Index query time comparison of large-scale dataset

Utilizing the initial 3,000,000 blocks from the large-scale dataset (Dataset I) on the public Ethernet network, this paper scrutinize the query response times of the EBTree and Dabble methods. The investigation encompasses scenarios where the number of master blocks ranges from 500 to 3,000 k, with a fixed number of slave blocks at 1,000. The experimental results are illustrated in Fig. 8.

As depicted in Fig. 8, the Multilevel Index (MLI) method demonstrates a 13.44% optimization in index construction time when compared to existing methods, particularly notable in large-scale datasets. This advantage becomes more pronounced as the number of blocks increases, surpassing the performance of the EBTree method.

**Figure 9:**
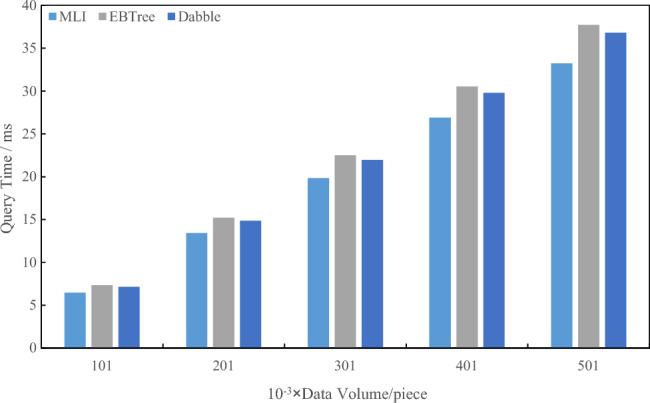
Index query time comparison of small-scale dataset.

Utilizing a small-scale dataset, namely Dataset 2: Lognormal artificial dataset, comprising 5 million data points with a total size of approximately 24 MB, this paper conducted a comparative analysis of the query response times between the EBTree and Dabble methods. Specifically, the comparison was made for scenarios involving the storage of 10, 20, 30, 40, and 500,000 pieces of data in the master block, along with 1,000 pieces of data in the slave block. The experimental results are presented in Fig. 9.

As depicted in Fig. 9, it is evident that the Multilevel Index (MLI) method achieves an optimization of approximately 10.71% in index construction time when compared to existing methods on small-scale datasets. The experimental outcomes underscore the superior query performance of the MLI method, particularly in scenarios where the data volume is substantial.

### Comparison of memory consumption

The weight matrix constructed by MLI in the slicing stage hardly takes up memory, and the master chain builds the index based on the jump consistent hash algorithm. Compared with the classical consistent hash, the jump consistent hash has almost no additional memory consumption, so the memory overhead in MLI mainly considers the index construction of the slave blockchain. The false-positive of IBF is set to 0.0137 bits. The EBTree method rewrites the blockchain structure, and the memory consumption is mainly blocked data. Therefore, this section will compare the Dabble method. The experimental results are shown in Table 2.

**Table 2:** Memory consumption comparison.

Method	Memory usage
Dabble	24 MB + 4 KB
MLI	24 MB + 2.048 KB

As can be seen from Table 2, the Lognormal data set takes up 24 MB of memory, while the neural network takes up 4 KB of memory in Dabble method, while the IBF in MLI still only takes up about 2.048 KB of memory within the allowable range of false positives. Even if IBF and BF are in the same false positives, these two methods can keep the same order of magnitude.

## Conclusions

As blockchain technology gains widespread application, the traditional single-chain structure has become inadequate to meet evolving demands. The incorporation of slave blockchains alongside a master chain expands the applicability of blockchain technology across various domains, such as education and food supply chains. This paper introduces a composite index construction method tailored for master–slave blockchains. Specifically, the entire master–slave blockchain structure undergoes segmentation based on the master chain, enhancing maintainability through the utilization of a weight matrix that supports the index construction. Building upon this foundation, index construction methods are proposed, addressing the distinct data scales between the master and slave chains. These methods leverage the Jump Consistency Hash algorithm for the master chain and an Improved Bloom Filter (IBF) for the slave chain, aiming to enhance query efficiency within master–slave blockchains. Experimental results demonstrate significant advantages in construction time, query efficiency, and memory consumption compared to existing methods.

This paper delves into an in-depth exploration of constructing multi-level indexes and optimizing consensus algorithms for master–slave blockchain structures. Proposed solutions address identified limitations of existing methods, yielding noteworthy research outcomes. Nevertheless, the intricate nature of the master–slave blockchain structure and the dynamic application scenarios present ongoing challenges. The following areas related to the paper's research content warrant further investigation:

1. In the index construction process, hash values are utilized for building node key-based indexes, yielding a noticeable advantage in construction time. However, to enhance query efficiency, exploring node key selection, such as incorporating semantic information based on application scenarios, remains a potential avenue for further research.
2. The node state transition mechanism currently relies solely on the consensus result as the scoring system benchmark. Future work will focus on optimizing the evaluation index for node behavior within this mechanism.

## Data Availability

The data used to support the findings of this study are available from the corresponding author upon request.

## References

[1] Barbosa, M. *et al.* SoK: Computer-aided cryptography. In *2021 IEEE Symposium on Security and Privacy (SP)* 777–795 (2021). 10.1109/SP40001.2021.00008.

[2] Moudoud, H., Cherkaoui, S. & Khoukhi, L. Towards a scalable and trustworthy blockchain: IoT Use Case. In *ICC 2021—IEEE International Conference on Communications* 1–6 (2021). 10.1109/ICC42927.2021.9500535.

[3] Li, C. *et al.* A decentralized blockchain with high throughput and fast confirmation. In *Proceedings of the 2020 USENIX Conference on Usenix Annual Technical Conference*, 515–528 (2020).

[4] Połap, D., Srivastava, G., Jolfaei, A. & Parizi, R. M. Blockchain Technology and Neural Networks for the Internet of Medical Things. In *IEEE INFOCOM 2020—IEEE Conference on Computer Communications Workshops (INFOCOM WKSHPS)* 508–513 (2020). 10.1109/INFOCOMWKSHPS50562.2020.9162735.

[5] Khan AA, Shaikh AA, Laghari AA (2023). IoT with multimedia investigation: a secure process of digital forensics chain-of-custody using blockchain hyperledger sawtooth. Arab. J. Sci. Eng..

[6] Ayub Khan, A., Laghari, A. A., Shaikh, Z. A., Dacko-Pikiewicz, Z. & Kot, S. Internet of Things (IoT) security with blockchain technology: A state-of-the-art review. *IEEE Access* **10**, 122679–122695 (2022).

[7] Khan AA (2022). A drone-based data management and optimization using metaheuristic algorithms and blockchain smart contracts in a secure fog environment. Comput. Electr. Eng..

[8] Khan AA, Laghari AA, Li P, Dootio MA, Karim S (2023). The collaborative role of blockchain, artificial intelligence, and industrial internet of things in digitalization of small and medium-size enterprises. Sci. Rep..

[9] Yin, M., Malkhi, D., Reiter, M. K., Gueta, G. G. & Abraham, I. HotStuff: BFT Consensus with linearity and responsiveness. In *Proceedings of the 2019 ACM Symposium on Principles of Distributed Computing* 347–356 (2019). 10.1145/3293611.3331591.

[10] Chen J, Micali S (2019). Algorand: A secure and efficient distributed ledger. Theor. Comput. Sci..

[11] Sui Y, Wang W, Deng X (2021). High throughput verifiable query method for blockchain-oriented off-chain database. J. Chin. Comput. Syst..

[12] Cai L, Zhu YC, Guo QX, Zhang Z, Jin CQ (2020). Efficient materialized view maintenance and trusted query for Blockchain. J. Softw..

[13] Wu H, Jiang S, Cao J (2023). High-efficiency Blockchain-based supply chain traceability. IEEE Trans. Intell. Transp. Syst..

[14] Xu H (2023). Empowering authenticated and efficient queries for STK transaction-based blockchains. IEEE Trans. Comput..

[15] Gao J, Cao X, Yao X, Zhang G, Wang W (2023). LMSFC: A novel multidimensional index based on learned monotonic space filling curves. Proc. VLDB Endow..

[16] XiaoJu, H., XueQing, G., ZhiGang, H., LiMei, Z. & Kun, G. EBTree: A B-plus tree based index for ethereum Blockchain data. In *Proceedings of the 2020 Asia Service Sciences and Software Engineering Conference* 83–90 (2020). 10.1145/3399871.3399892.

[17] Kipf, A. *et al.* RadixSpline: a single-pass learned index. In *Proceedings of the Third International Workshop on Exploiting Artificial Intelligence Techniques for Data Management* 1–5 (2020). 10.1145/3401071.3401659.

[18] Xing X, Chen Y, Li T, Xin Y, Sun H (2021). A blockchain index structure based on subchain query. J. Cloud Comput..

[19] Alghamdi, N., Zhang, L., Zhang, H., Rundensteiner, E. A. & Eltabakh, M. Y. ChainLink: Indexing Big Time Series Data For Long Subsequence Matching. in *2020 IEEE 36th International Conference on Data Engineering (ICDE)* 529–540 (2020). 10.1109/ICDE48307.2020.00052.

[20] Gao YN, Ye JB, Yang NZ, Gao XF, Chen GH (2020). Middle layer based scalable learned index scheme. J. Softw..

